# Optimizing Intracerebral Hemorrhage Management and Interhospital Transfer With Viz ICH Plus AI Technology

**DOI:** 10.7759/cureus.80790

**Published:** 2025-03-18

**Authors:** Ryan Afreen, Bahie Ezzat, Roshini Kalagara, Neha S Dangayach, Christopher P Kellner

**Affiliations:** 1 Neurosurgery, Icahn School of Medicine at Mount Sinai, New York, USA

**Keywords:** artificial intelligence (ai), interhospital transfer, intracerebral hemorrhage, stroke management, viz.ai, viz ich plus

## Abstract

This case study explores the integration of Viz ICH Plus, an AI-powered intracerebral hemorrhage (ICH) detection system, into a centralized program called the Neuroemergencies Management and Transfer (NEMAT) program of a large urban healthcare system. The study highlights how Viz ICH Plus promptly identified a right parieto-occipital hematoma in a patient presenting with a headache, resulting in a marked reduction in interhospital transfer (IHT) time. The patient underwent a successful supratentorial craniotomy for hematoma evacuation and demonstrated significant cognitive and physical improvement over the following year. Viz ICH Plus reduced IHT time from approximately 200 to 101 minutes, expediting access to definitive care and improving patient outcomes. Standard of care radiology review of the scan and communication of results could have added to additional delays in transferring this patient to receive definitive care. This case study illustrates a substantial reduction in transfer time and highlights the potential of AI to transform stroke care by optimizing response times and facilitating timely interventions.

## Introduction

Intracerebral hemorrhage (ICH) comprises 10-15% of all strokes and is associated with high morbidity and mortality [1]. With a 40% fatality rate within one month of onset, only 12-39% of patients regain long-term functional independence [2,3]. Management of ICH is complex and dynamic, shaped by ongoing research that influences decisions on medical versus surgical interventions and their timing. Early Minimally Invasive Removal of Intracerebral Hemorrhage (ENRICH) was the first large randomized controlled trial to demonstrate a functional benefit of early surgical evacuation within 24 hours, marking a significant paradigm shift in ICH treatment protocols [4]. ENRICH highlighted the evolving landscape of ICH management and the critical role of prompt treatment [4].

Interhospital transfer (IHT) poses an interesting challenge, as several prior studies have highlighted the potential for delays in care in various clinical situations [5-8]. This issue is critical in cases requiring specialized treatment, such as ICH evacuation, where there may be a single facility in a hospital system uniquely optimized for management. Taken together, the ability to streamline care for patients with ICH through rapid transfer and treatment is of utmost interest to patients, care providers, and hospital systems. As such, the recent implementation of AI programs into medical care presents a potent solution, as they may streamline and optimize several steps in the care pathway. This process involves patient identification, approval of the receiving center, and transportation to the final facility. Viz ICH Plus, a 510(k)-cleared AI-powered detection system, holds particular utility in these efforts for ICH patients for its capability to automate the process of hemorrhage detection [9-11].

In this case study, we report on our AI-guided stroke detection program to streamline IHT and decrease the time to treatment in the management of ICH. Specifically, by utilizing Viz ICH Plus to assess all CT scans automatically, we will be able to promptly identify potential hemorrhages and alert the transferring and receiving hospitals’ corresponding stroke, neurosurgical, and neurocritical care team members. This reduces the time to patient identification and the activation of downstream processes for rapid and safe triage and transfer.

## Case presentation

To evaluate the effectiveness of Viz ICH Plus on IHT for ICH patients, three main metrics were assessed: the time from patient identification at the transferring center to alerting the receiving center, the duration from hemorrhage identification to patient arrival at the receiving center, and the overall patient outcome.

**Figure 1:**
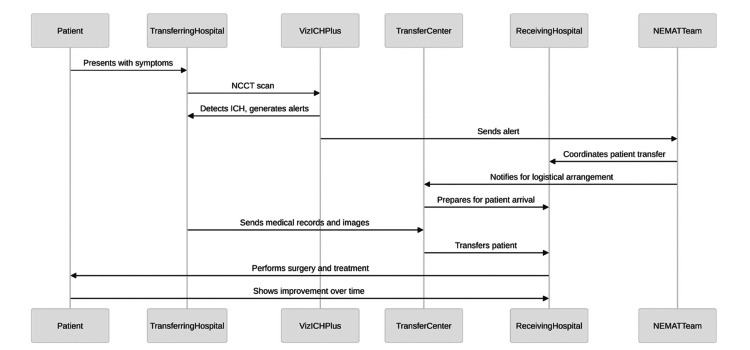
Process map of the NEMAT system sequence of operations from ICH presentation through treatment and follow-up streamlined by Viz ICH Plus ICH, intracerebral hemorrhage; NEMAT, Neuroemergencies Management and Transfer Image credit: This figure was created by Bahie Ezzat using the R programming language.

**Figure 2:**
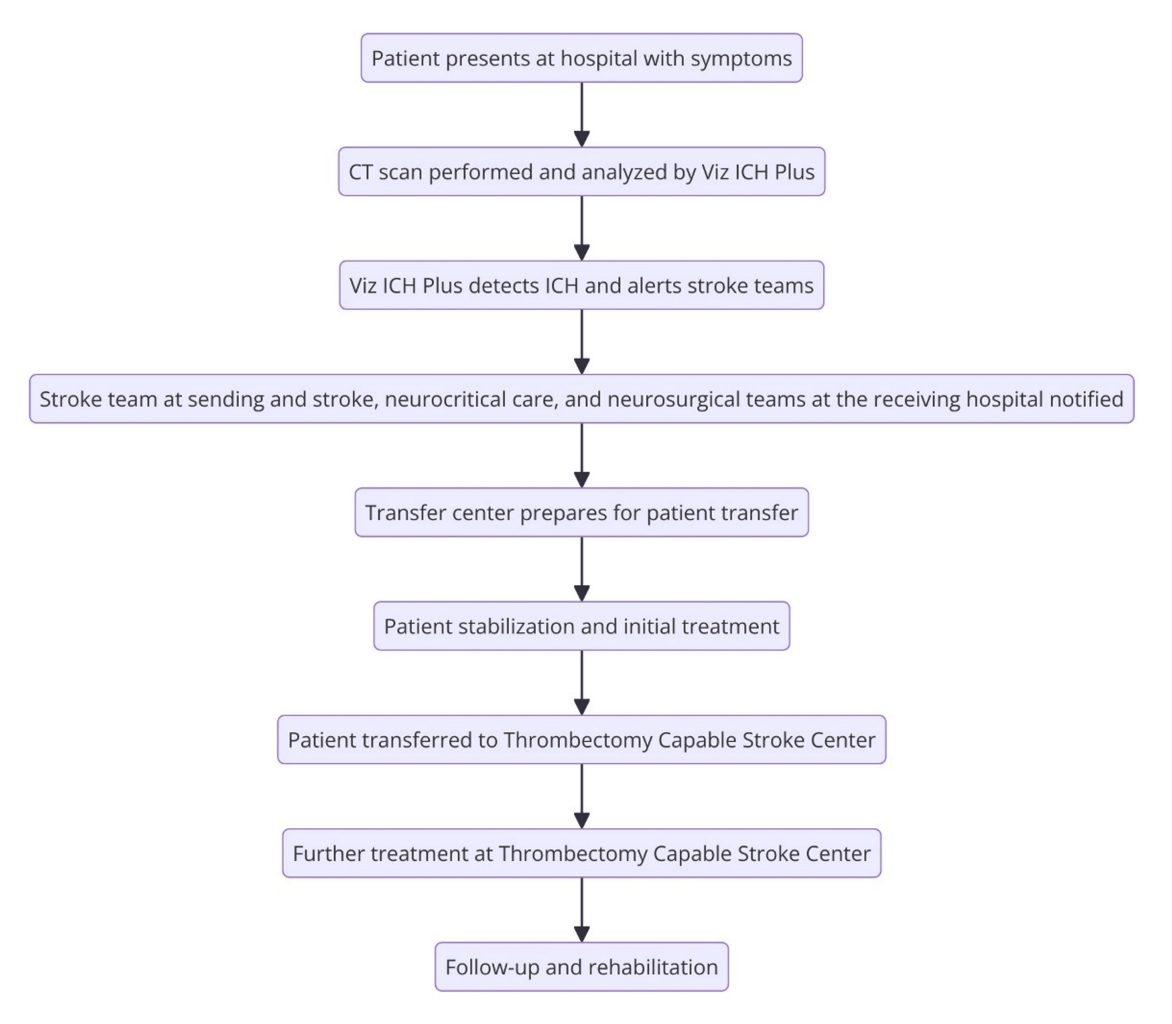
Flowchart of ICH patient management process from initial presentation to rehabilitation using Viz ICH Plus for rapid ICH detection and team coordination ICH, intracerebral hemorrhage Image credit: This figure was created by Bahie Ezzat using the R programming language.

In this case study (Table 1), a 47-year-old male with a past medical history of hypertension presented at the transferring hospital with a severe headache, nausea, and vomiting but no speech or motor deficits. Viz ICH Plus analyzed the patient’s CT, revealing a substantial right parieto-occipital hematoma with ventricular extension, and automatically alerted the Neuroemergencies Management and Transfer (NEMAT) team at the receiving center (Figure 1). NEMAT specializes in the expedited transfer and management of neurological emergencies through improved transfer protocols [12]. After reviewing CT scans, the team coordinated with the receiving hospital through a centralized command center, informed the ED provider, and recommended initial stabilization measures, including clevidipine and levetiracetam administration. With an ICH score of 2, the patient was transferred within 101 minutes across a 13.7-mile distance - much faster than the average IHT time of 199.7 minutes for similar cases (Figure 2) [13]. Without Viz ICH Plus, this patient’s care would have been dependent on a radiologist’s read of the CT of the head and notification of the frontline ED staff and stroke team staff. Then, the ED and stroke team members would call the command center to notify them of the potential need for transfer. By directly notifying specialist teams, we were able to save time and improve the efficiency of diagnosis and the transfer process.

**Table 1:** Case study patient information ICH, intracerebral hemorrhage; IHT, interhospital transfer; mRS, modified Rankin Scale; NIHSS, National Institutes of Health Stroke Scale; VP, ventriculoperitoneal

Category	Details
Patient demographics	47-year-old male
Chief complaint	Severe headache, nausea, and vomiting; no speech or motor deficits
Past medical history	Hypertension
Initial presentation	Presented at the transferring hospital with symptoms of ICH
Imaging findings	CT scan showed a substantial right parieto-occipital hematoma with ventricular extension
Intervention	Underwent supratentorial craniotomy with endoscopic hematoma evacuation and VP shunt placement
Transfer details	Transferred within 101 minutes across a 13.7-mile distance (faster than the average IHT time of 199.7 minutes)
Outcome and follow-up	ICU stay: eight days; total hospital stay: 19 days; gradual cognitive and memory improvement; one-year follow-up showed no recurrent ICH, stable catheter positioning, mRS 1, NIHSS 2

Outcome and follow-up

Upon arrival, the patient underwent a supratentorial craniotomy with endoscopic hematoma evacuation and ventriculoperitoneal shunt placement. The time from the initial bleed to surgery was 30.6 minutes. The patient’s ICU stay was eight days, with a total hospital stay of 19 days before discharge. One month later, the patient’s modified Rankin Scale (mRS) and National Institutes of Health Stroke Scale (NIHSS) scores were 4 and 6, respectively, reflecting cognitive and memory improvements. By three months, the patient’s scores improved to 3 and 5. Six- and 12-month follow-up CT scans showed no recurrent ICH or infarct, with stable catheter positioning. In one year, his scores had further improved to mRS 1 and NIHSS 2.

## Discussion

Our large urban healthcare system adopted an innovative AI tool, Viz ICH Plus, to support the current NEMAT system with expedited intervention for an ICH patient requiring IHT. This strategic integration into existing transfer protocols, administered through the NEMAT program, aimed to substantially decrease key transfer metrics for confirmed cases of ICH requiring transportation to a specialized stroke center. The objective underlying the accelerated diagnosis and team notification was to considerably lower the critical time between identification and treatment.

Existing research indicates a correlation between critically ill patients undergoing IHT and longer hospital stays [14]. To counteract these clinical outcomes, Viz ICH provides rapid stroke detection and triage critical cases, as exemplified in this case study, facilitating reduced IHT time through streamlined communication, transfer prioritization, and remote access to specialized expertise. For example, a study in the American Journal of Neuroradiology found that Viz.Ai reduced text messaging thread counts by 30%, thus significantly improving communication in a comprehensive stroke center [15]. Another Viz.Ai implementation research study indicated a median transfer time reduction by an average of 22.5 minutes from CT angiography at a primary stroke center to door-in at a comprehensive stroke center [16]. In addition to large healthcare systems, Viz ICH Plus can also enhance stroke code workflows in settings with limited access to neuroradiologists or vascular neurologists [17]. Successful integration into hospital systems involves infrastructure compatibility to ensure compatibility with the AI model, a functional IHT system to manage triage and transfer protocols efficiently, and team coordination between the AI model and medical teams at both transferring and receiving hospitals.

Integration of AI tools like Viz ICH Plus, however, raises ethical and technical concerns. Algorithmic bias and validation challenges remain significant issues, as AI models may not generalize well across diverse patient populations or imaging protocols, potentially leading to disparities in care [18]. Additionally, ensuring transparency, data privacy, and accountability in AI-driven clinical decision-making is crucial, as reliance on machine-generated analyses without human oversight may introduce risks related to misdiagnosis and patient safety [18]. One of the unique challenges we encountered utilizing Viz ICH Plus in streamlining ICH detection was the variability in CT scanner technologies and imaging protocols across different facilities. Each hospital had a unique set of hardware from various manufacturers, which often required custom configuration to meet the specific image analysis requirements of Viz ICH Plus. This variability sometimes limited the AI program’s ability to view and assess certain scans, particularly those that did not conform to the preferred resolution and slice parameters. Another major challenge that emerged as a technical bottleneck was the bandwidth limitation in exporting all DICOM CT scans to Viz ICH Plus. The sheer volume of data processed during peak periods strained our existing IT infrastructure, occasionally delaying the transfer of critical imaging data to Viz ICH Plus for analysis. This issue underscored the need for robust system upgrades to enhance data-handling capacities, which may involve significant investment and coordination with our IT departments.

Despite the few technical and logistical challenges encountered within our system, the benefits of using Viz ICH Plus in managing ICH far outweigh its limitations. In addition to reducing IHT time, Viz ICH Plus enhances clinical workflow efficiency by providing more accurate hematoma volume estimation and streamlining IHT, ultimately reducing the time to treatment compared to manual methods. For example, Viz.Ai implemented at a large healthcare system showed that the mean volume difference between the semi-autonomous segmentation (SAS) ground truth and Viz.Ai was significantly smaller (4.77 ± 4.06 mL) compared to the mABC/2 method (8.36 ± 9.48 mL) (p < 0.01), which was measured by a board-certified neurosurgeon [19]. This indicates that Viz.Ai offers a more precise hematoma volume estimation, which is critical for clinical decision-making and patient management [19]. This study also reported that Viz.Ai’s average time-to-volume calculation was 151 ± 49.7 seconds, compared to 424 ± 208 seconds for SAS (p < 0.01) [19]. By reducing the time required for volume calculation, Viz.Ai improves clinical workflow efficiency [19]. So far, the Viz platform has been implemented across 97 hospitals in 20 US states, utilizing data from 23,223 patients [20].

AI solutions, such as Viz ICH Plus, can significantly decrease treatment time with automated pathology detection on imaging. The ENRICH trial demonstrated that faster interventions were valuable in urgent clinical scenarios and associated with improved functional outcomes [4]. Expanding the use of AI in such care pathways will be crucial for improving metrics such as time from initial presentation to patient identification, duration from ictus to treatment, and overall patient outcomes.

## Conclusions

This case study illustrates how AI software can expedite treatment for ICH patients necessitating IHT to an ICH-focused stroke center. With its unparalleled ability to analyze all imaging related to stroke codes, Viz ICH Plus promptly identified and alerted stroke team members at both the transferring and receiving hospitals, ensuring efficient and timely care. This case also highlights the effectiveness of AI in managing ICH cases requiring IHT. By streamlining ICH detection, Viz ICH Plus expedites coordination between hospitals and healthcare teams, thus reducing time to treatments and enhancing stroke care.

While AI tools like Viz ICH Plus enhance efficiency in stroke care, their successful implementation requires infrastructure compatibility, ongoing validation, and integration with existing clinical workflows. Additionally, careful consideration must be given to algorithmic bias, imaging standardization, and data security to ensure equitable and reliable patient care. Despite these challenges, our findings reinforce that AI-driven solutions are an invaluable asset in stroke management, particularly in reducing delays associated with IHT and accelerating access to specialized stroke centers. Continued refinement and broader adoption of AI in neurosurgical workflows have the potential to further optimize stroke care and improve patient outcomes on a larger scale.
